# Molecular basis for the evolved instability of a human G-protein coupled receptor

**DOI:** 10.1016/j.celrep.2021.110046

**Published:** 2021-11-23

**Authors:** Laura M. Chamness, Nathan B. Zelt, Haley R. Harrington, Charles P. Kuntz, Brian J. Bender, Wesley D. Penn, Joshua J. Ziarek, Jens Meiler, Jonathan P. Schlebach

**Affiliations:** 1 Department of Chemistry, Indiana University, Bloomington, IN 47405, USA; 2 Department of Molecular and Cellular Biochemistry, Indiana University, Bloomington, IN 47405, USA; 3 Department of Chemistry, Vanderbilt University, Nashville, TN 49795, USA; 4 Institut for Drug Development, Leipzig University, Leipzig, SAC, Germany; 5 Lead contact

## Abstract

Membrane proteins are prone to misfolding and degradation. This is particularly true for mammalian forms of the gonadotropin-releasing hormone receptor (GnRHR). Although they function at the plasma membrane, mammalian GnRHRs accumulate within the secretory pathway. Their apparent instability is believed to have evolved through selection for attenuated GnRHR activity. Nevertheless, the molecular basis of this adaptation remains unclear. We show that adaptation coincides with a C-terminal truncation that compromises the translocon-mediated membrane integration of its seventh transmembrane domain (TM7). We also identify a series of polar residues in mammalian GnRHRs that compromise the membrane integration of TM2 and TM6. Reverting a lipid-exposed polar residue in TM6 to an ancestral hydrophobic residue restores expression with no impact on function. Evolutionary trends suggest variations in the polarity of this residue track with reproductive phenotypes. Our findings suggest that the marginal energetics of cotranslational folding can be exploited to tune membrane protein fitness.

## In brief

Integral membrane proteins are prone to misfolding, especially mammalian gonadotropin-releasing hormone receptors (GnRHRs). Chamness et al. show that the evolved instability of mammalian GnRHRs stems from adaptive modifications that disrupt translocon-mediated membrane integration, suggesting that membrane protein misfolding can be exploited to tune fitness.

## Graphical abstract

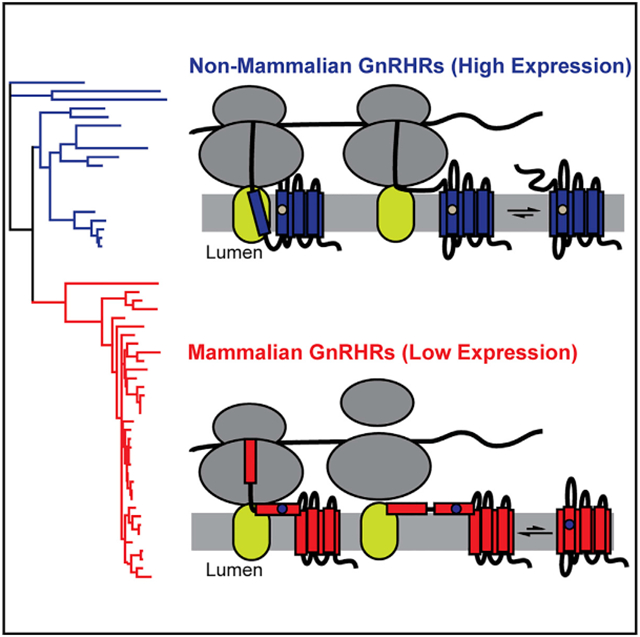

## INTRODUCTION

Proteins must continually sample new mutations that modify their conformational equilibria in order to maximize their evolutionary fitness (Harms and Thornton, 2013). Most mutations destabilize native protein structures, and evolutionary pathways are limited to those in which the protein retains sufficient conformational stability and activity with each successive mutation (Tokuriki and Tawfik, 2009). Like water-soluble proteins, the sequences of integral membrane proteins (MPs) are also constrained by folding energetics (Marinko et al., 2019). However, their transmembrane (TM) domains must also retain sufficient hydrophobicity to partition into the membrane, which further constrains their evolutionary sequence space (Marinko et al., 2019). In recent investigations of the sequence constraints within the class A G-protein coupled receptor (GPCR) rhodopsin, we found its expression to be highly sensitive to mutations within a marginally hydrophobic TM domain (Roushar et al., 2019). Moreover, the natural sequence of this TM domain appears to be more polar than is necessary to support function, and its expression can be enhanced by functionally neutral hydrophobic substitutions (Roushar et al., 2019). As a result of this instability, much of the nascent protein is retained in the endoplasmic reticulum (ER) (Roushar et al., 2019). As is true for water-soluble proteins, these observations suggest that naturally evolved MPs tend to be metastable and have not evolved to maximize the efficiency of protein biogenesis. Nevertheless, it is unclear whether this metastability provides an evolutionary benefit or if it is simply an emergent property that stems from the evolutionary process itself.

A series of previous investigations of the gonadotropin releasing hormone receptor (GnRHR), another class A GPCR, revealed that the mammalian forms of these receptors exhibit a heightened tendency to misfold and accumulate within the ER (Janovick et al., 2003). GnRHR plays a critical role in steroidogenesis, and its functional expression at the plasma membrane is critical for reproductive fitness (Janovick et al., 2006, 2013). Variations within the *GnRHR* gene have been associated with shifts in litter size and length of the luteal phase (Bemji et al., 2018; Conn et al., 2006b; Ulloa-Aguirre et al., 2006; Yang et al., 2011). Additionally, numerous loss-of-function mutations in human *GnRHR* have been found to cause hypogonadotropic hypogonadism (HH), which is characterized by infertility and loss of gonadal function (Janovick et al., 2013). GnRHRs found in fish, which produce many offspring, appear to exhibit robust plasma membrane expression (PME) relative to those found in mammals, which have far fewer offspring (Janovick et al., 2006; Conn et al., 2006b). Based on various observations, it has been suggested that these reproductive selection pressures increased the relative fitness of mammals expressing less stable GnRHR variants with diminished PME (Ulloa-Aguirre et al., 2006; Conn et al., 2006a). It has also been speculated that the pool of immature GnRHRs in the ER may provide a regulatory benefit because modifications to the proteostasis network can alter the flux of mature protein through the secretory pathway (Janovick et al., 2006; Conn et al., 2006b; Conn and Ulloa-Aguirre, 2011). Thus, it appears as though nature may have exploited the instability of GnRHRs in order to tune their evolutionary fitness. Nevertheless, the nature of the conformational modifications involved in these evolutionary adaptations remains poorly understood.

To evaluate the evolutionary sequence modifications that coincide with the proteostatic divergence between the mammalian and non-mammalian GnRHRs, we first confirm that a C-terminal truncation within the mammalian receptors appears to significantly contribute to the attenuation of the mammalian receptor PME and show that this modification compromises the membrane integration of TM7. Additionally, we demonstrate that the TM domains of mammalian GnRHRs are considerably more polar than those of non-mammalian GnRHRs, and that these modifications also compromise the translocon-mediated membrane integration of two TM domains. Structural models of these receptors suggest two of the polar substitutions that compromise translocon-mediated membrane integration of the nascent chain occur at surface residues that are projected into the membrane core. Moreover, we show that re-introducing the ancestral hydrophobic side chain at one of these positions partially restores the PME of human GnRHR with minimal impact on receptor activation. Finally, we show that natural variations in the polarity of these residues among mammalian GnRHRs are associated with dramatic variations in litter size. Together, these findings provide evidence that evolution has exploited the marginal cotranslational folding energetics of GnRHR in order to tune its fitness. More generally, these observations suggest the instability of natural proteins provides an additional avenue for evolutionary adaptation.

## RESULTS

### Cellular expression of natural GnRHRs

**Figure 1.**
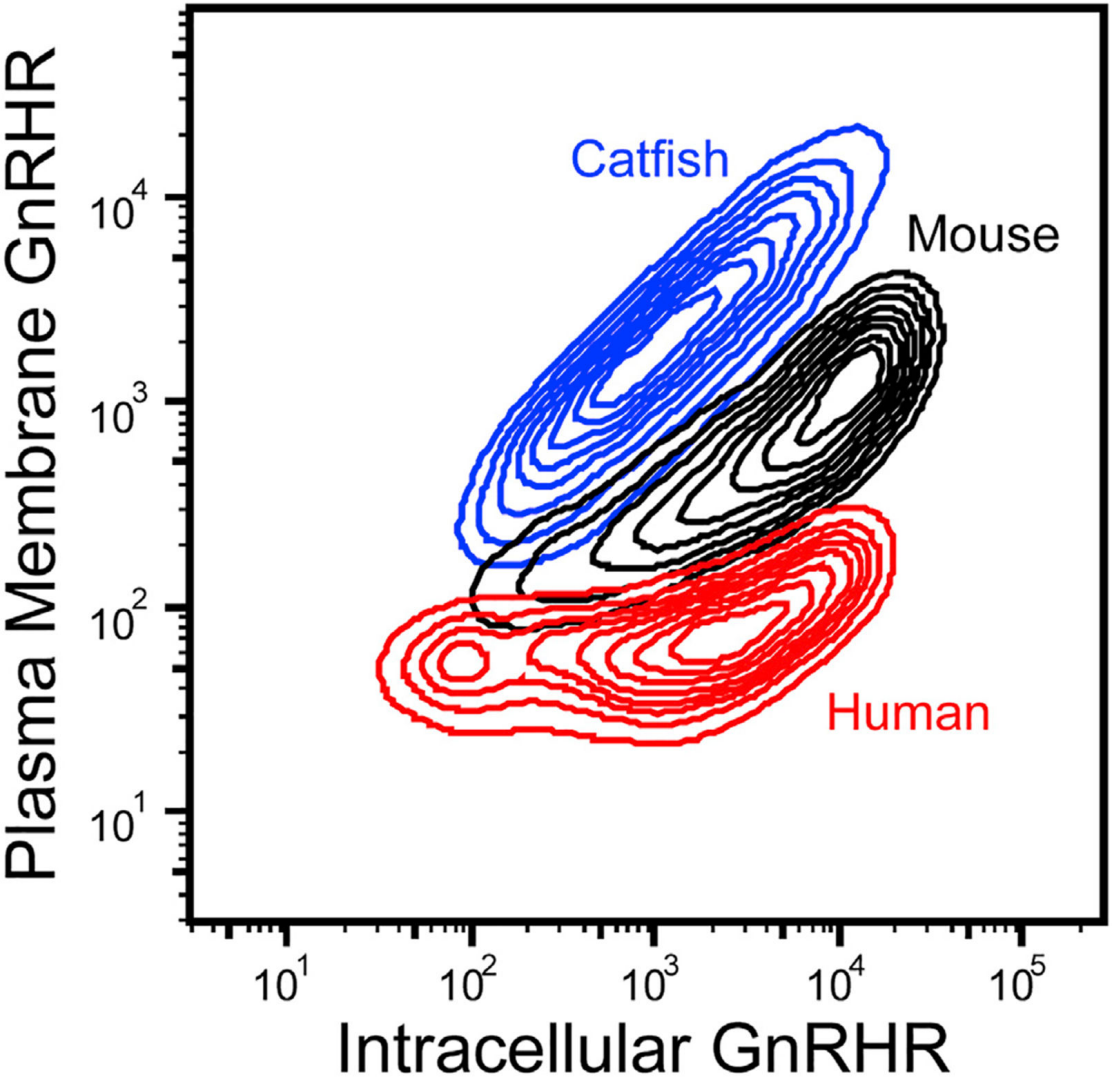
Cellular trafficking of GnRHR variants in HEK293T cells Human (red), mouse (black), and catfish (blue) GnRHRs were transiently expressed in HEK293T cells, and the relative abundance of plasma membrane GnRHR and intracellular GnRHR was analyzed by flow cytometry. Contour plots show the distribution of cellular fluorescence intensities associated with immunostaining of plasma membrane (y coordinate) and intracellular (x coordinate) GnRHRs for one representative biological replicate.

Various lines of evidence suggest the selection for reduced GnRH signaling in higher mammals produced GnRHRs with diminished conformational stability and attenuated plasma membrane expression (PME) (Janovick et al., 2003; Lin et al., 1998). Nevertheless, most previous studies relied on activity as a proxy for PME (Janovick et al., 2006; Conn et al., 1987; Leaños-Miranda et al., 2003). To more directly probe differences in GnRHR expression, we employed immunostaining in conjunction with flow cytometry to quantitatively characterize the expression of three previously characterized GnRHRs (human, mouse, and catfish). Briefly, each of these receptors was transiently expressed in HEK293T cells prior to labeling plasma membrane and intracellular GnRHRs with distinct fluorescent antibodies, as previously described (Schlebach et al., 2015). Cellular fluorescence profiles were then analyzed by flow cytometry. A comparison of the distribution of single-cell fluorescence profiles reveals that larger proportions of the expressed mouse GnRHR (*Mus musculus*, mGnRHR) and catfish GnRHRs (*Clarius gariepinus*, cGnRHR) accumulate at the plasma membrane relative to human GnRHR (*Homo sapiens*, hGnRHR) (Figure 1). The mean fluorescence intensity associated with the surface immunostaining of hGnRHR at the plasma membrane is 21.5- ± 6.0-fold lower than that of mGnRHR and 92.0- ± 17.9-fold lower than that of cGnRHR. Overall, the total cellular expression of hGnRHR was 2.71- ± 0.04-fold lower than that of mGnRHR and 2.04- ± 0.35-fold lower than cGnRHR. These results directly show that cGnRHR exhibits robust expression and trafficking relative to the mammalian receptors under equivalent conditions. Nevertheless, the nature of the structural modifications responsible for this apparent proteostatic divergence remains unclear.

### Impact of the C-terminal tail in GnRHR expression and topology

**Figure 2.**
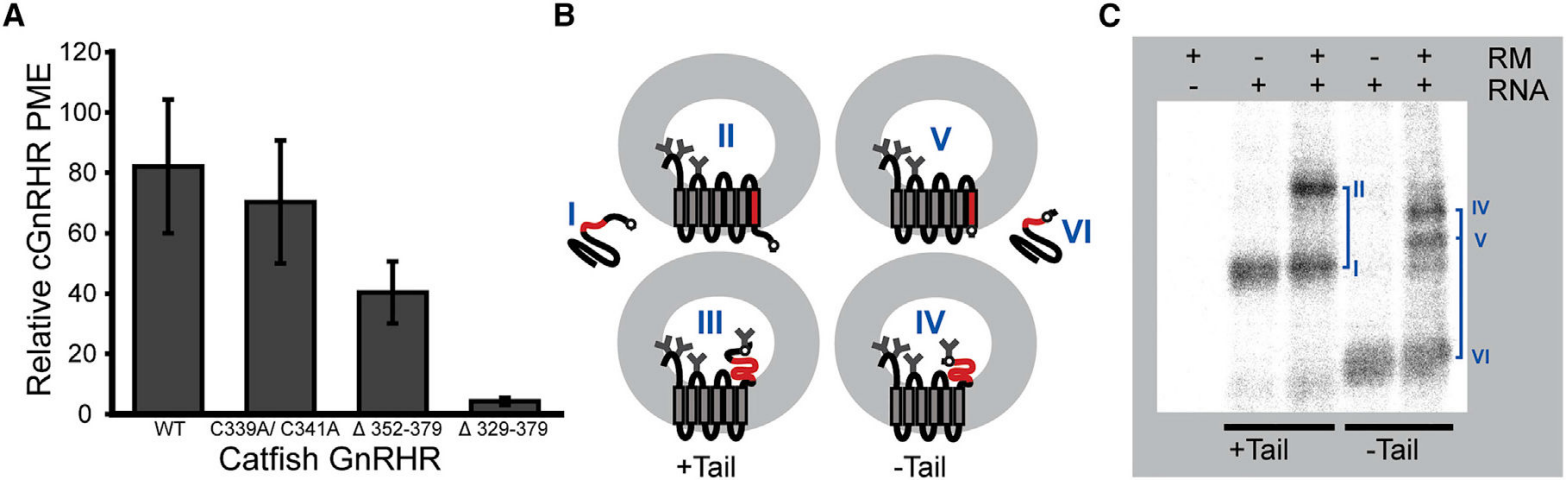
Impact of C-terminal modifications on the plasma membrane expression and topology of catfish GnRHR (A) Catfish GnRHRs bearing various C-terminal modifications were transiently expressed in HEK293T cells prior to analysis of surface immunostaining of plasma membrane GnRHR by flow cytometry. A bar graph depicts the mean fluorescence intensity associated with the surface immunostaining of a series of catfish GnRHR variants normalized relative to that of human GnRHR. Values reflect the average of three biological replicates, and error bars reflect the standard deviation. (B) A cartoon illustrates how the glycosylation state of full-length (+ tail) and truncated (− tail) catfish GnRHR variants bearing a C-terminal glycosylation site varies with topology. In each case, a failure of TM7 to undergo translocon-mediated membrane integration results in the incorporation of an additional glycan. The topologies of the glycoforms are assigned roman numerals for reference. (C) A representative SDS-PAGE image shows catfish GnRHR variants containing a C-terminal glycosylation site, translated in canine rough microsomes. Negative control reactions lacking RNA (first lane) or containing RNA but lacking microsomes (second and fourth lanes) are shown for reference. The glycoform for each corresponding band is indicated for reference in blue. See also Figure S1.

Evolutionary adaptations in mammalian GnRHRs coincided with a variety of sequence modifications. Most strikingly, mammalian GnRHRs feature a C-terminal deletion of a disordered loop as well as a conserved amphipathic helix (helix 8 [H8]) that contains two palmitoylation sites (Lin et al., 1998; Millar, 2005; Palczewski et al., 2000). Fusing the C-terminal domain of cGnRHR to the C terminus hGnRHR was previously shown to enhance the activity of the human receptor (Janovick et al., 2003). Nevertheless, the extent to which the structural elements within the C-terminal region impact the PME remains unclear. We therefore assessed the effects of various C-terminal modifications on the PME of cGnRHR. To determine whether C-terminal palmitoylation impacts PME, we first characterized a double mutant of cGnRHR that lacks its two C-terminal palmitoylation sites (C339A and C341A). Removal of these palmitoylation sites has minimal effect on the PME of cGnRHR (Figure 2A), which suggests the loss of these post-translational modifications is not responsible for the attenuated PME of the mammalian receptors. To determine whether the disordered portion of the C-terminal tail impacts PME, we next characterized a cGnRHR variant with a deletion downstream of H8 (Δ352-379). Truncation of these residues reduces the PME of cGnRHR 2.0- ± 0.2-fold (Figure 2A), which suggests this portion of the tail is important for efficient expression. Finally, to determine whether H8 impacts PME, we characterized a cGnRHR variant lacking the entire C-terminal tail (Δ329-379), which mimics the deletion found in mammalian receptors (Millar, 2005). This truncation reduces the PME of cGnRHR 19.4- ± 0.9-fold (Figure 2A), which demonstrates that the truncation of H8 also contributes to the diminished PME of mammalian GnRHRs. Nevertheless, our findings suggest that this change in PME cannot be directly linked to the loss of a single specific structural feature within the C-terminal tail.

Previous investigations have concluded that evolutionary modifications of PME arise from variations in the conformational stability of GnRHR (Janovick et al., 2003; Ulloa-Aguirre et al., 2006). Such variations should alter the propensity of the receptor to misfold during translocon-mediated cotranslational folding (stage I) and/or post-translational folding (stage II) (Popot and Engelman, 1990). Interestingly, Sun and Mariappan (2020) recently found that translocon-mediated membrane integration of C-terminal TM domains, which is the final step of stage I folding, becomes inefficient when the C-terminal loop is shorter than ~60 amino acids. In this case, translation terminates before the final TM domain can reach the translocon. To assess whether the C-terminal truncation of GnRHR compromises topogenesis, we utilized a glycosylation-based topology reporter to assess the effects of the tail on the orientation of TM7. Briefly, we introduced a consensus N-linked glycosylation site near the C terminus of cGnRHR, and then generated a second version also containing this C-terminal glycosylation site but lacking the tail. Formation of the native topology should result in the efficient glycosylation of three native lumenal sites while precluding the glycosylation of the C-terminal site. Alternatively, the misincorporation of TM7 should append an additional glycan at the C terminus (Figure 2B).

*In vitro* translation of the full-length receptor in the presence of canine rough microsomes produces two bands—one low weight band corresponding to the untargeted/unglycosylated protein (band I) and a higher weight band bearing the native glycans (band II) (Figure 2C). In contrast, truncation of the C-terminal tail generates three bands that correspond to the untargeted/unglycosylated protein (band VI), a higher weight band bearing the native glycans (band V), and a band bearing an additional glycan resulting from the misintegration of TM7 (band IV) (Figure 2C). Consistent with the findings of Sun and Mariappan (2020), the appearance of an additional high weight band upon truncation of the C-terminal tail suggests this natural modification indirectly compromises the topology of nascent GnRHR. Taken together, our results suggest the evolutionary truncation of the C terminus of mammalian GnRHRs compromises the fidelity of topogenesis in a manner that coincides with a sizable decrease in PME.

### Impact of sequence variations on the topological energetics of GnRHR

Although the C-terminal truncation of GnRHR appears to coincide with a substantial decrease in expression, the PME of truncated cGnRHR variant is still 4.2- ± 1.1-fold higher than that of hGnRHR. Moreover, the PME of the mouse receptor is still 21.5- ± 6.0-fold higher than that of the human receptor even though they both lack the C-terminal tail. Based on this consideration, we suspected that additional sequence modifications have tuned the PME among mammalian GnRHRs. Outside of the topogenic constraints associated with C-terminal TM domains, the efficiency of stage I folding primarily depends on the hydrophobicity of TM domains and the corresponding energetics of translocon-mediated membrane integration (Hessa et al., 2005). To assess how sequence modifications may have impacted the fidelity of stage I folding, we analyzed the sequences of cGnRHR and hGnRHR using a knowledge-based algorithm that predicts the free energy difference associated with the transfer of nascent TM domains from the translocon to the ER membrane (ΔG predictor) (Hessa et al., 2007). A scan of the cGnRHR sequence reveals that its first six of its TM domains have pronounced energetic minima (TM7 is quite polar), four of which have negative transfer-free energies (Figure S1). This observation suggests that most TM domains within cGnRHR are sufficiently hydrophobic to undergo efficient translocon-mediated membrane integration. By comparison, only two of seven TM domains within hGnRHR have negative transfer-free energies (Figure S1), which suggests this protein may be more prone to the formation of topological defects during stage I folding of its first six TM domains.

**Figure 3.**
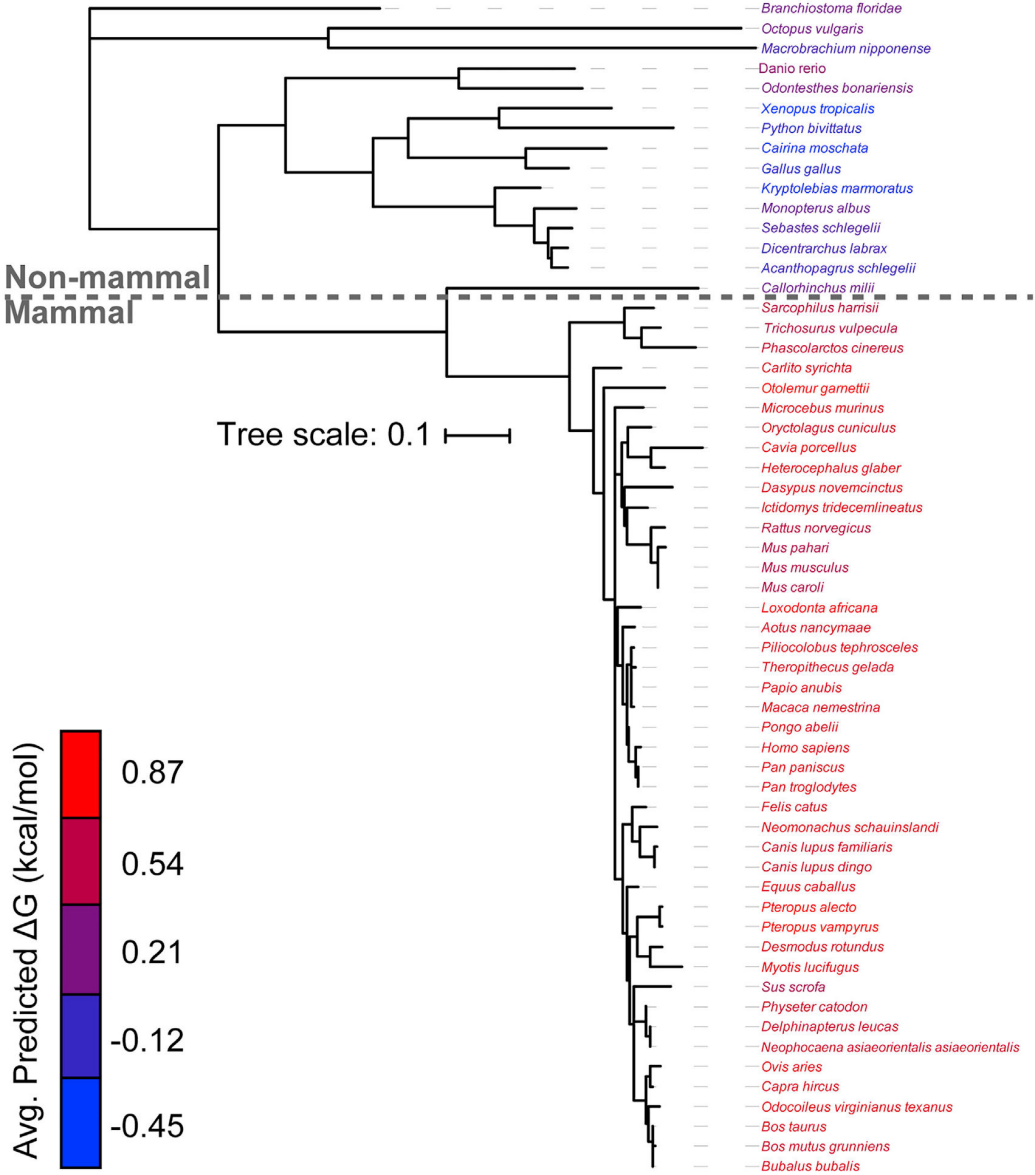
Evolutionary divergence of the topological energetics of GnRHR The predicted transfer-free energy associated with the translocon-mediated membrane integration of each TM domain within 59 known GnRHRs was calculated using the ΔG predictor (Hessa et al., 2007), and the average value for the seven TM domains within each receptor was projected onto a phylogenetic tree. The names of each species within the phylogenetic tree are colored according to the average ΔG value for the seven TM domains within the corresponding receptor. The phylogenetic tree was constructed using ML after MUSCLE alignment using MEGA7 software, and the branch lengths represent number of substitutions per site. A length unit of 0.1 substitutions per site corresponds to 10% divergence. The *Clarius gariepinus* (catfish) receptor is annotated as a type II GnRHR and was therefore excluded from this analysis (see STAR Methods). See also Table S1.

**Figure 4.**
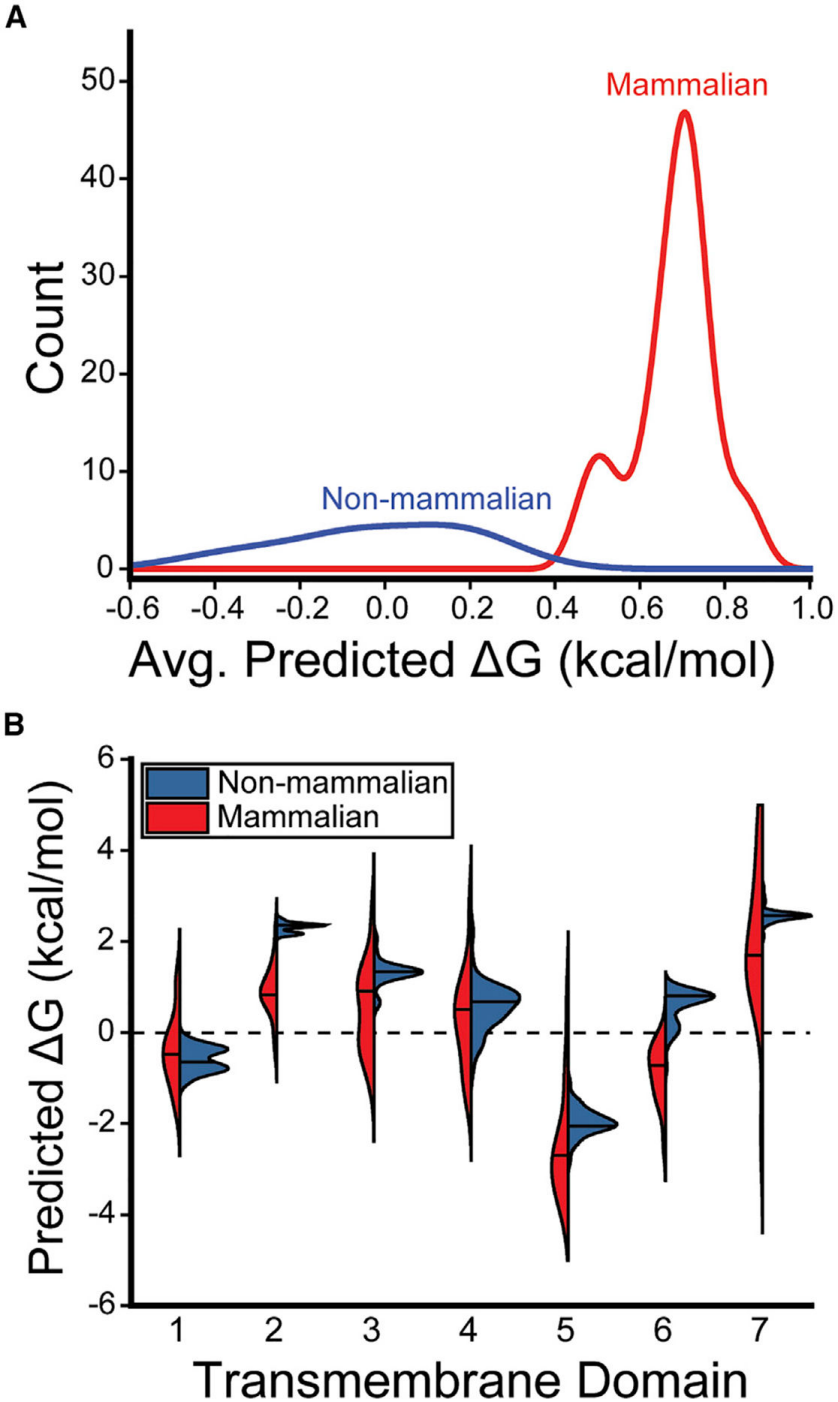
Comparison of the topological energetics of mammalian and non-mammalian GnRHRs The distribution of predicted transfer-free energies associated with the translocon-mediated membrane integration of the TM domains within 59 known GnRHRs are shown. (A) A histogram depicts the distribution of the average transfer-free energies across the seven TM domains of the non-mammalian (blue) and mammalian (red) GnRHRs. (B) A series of violin plots depict the distribution of predicted transfer-free energies for each individual TM domain found within the non-mammalian (blue) and mammalian (red) receptors. The position of the median value is indicated by a horizontal line within each distribution. The shapes of the histograms and violins were generated using a kernel smoothing function. See also Table S1.

To determine whether these differences are reflective of a wider evolutionary trend, we used the ΔG predictor to scan the sequences of a total 59 known GnRHRs (Table S1). Projection of the average predicted transfer-free energies across the seven TM domains of each receptor onto a phylogenetic tree reveals stark contrasts in the topological energetics of mammalian and non-mammalian GnRHRs (Figure 3). The average predicted transfer-free energies are significantly higher among mammalian GnRHRs relative to those of the non-mammalian receptors (Figure 4A, Mann-Whitney p = 5 × 10^−14^). A comparison of the distribution of predicted transfer-free energies for individual domains reveals that evolutionary adaptations resulted in particularly stark increases in the polarity of TM2 and TM6 (Figure 4B). It is unclear how sequence modifications within these domains may have impacted the energetics of post-translational folding reactions or functional signaling. Nevertheless, heightened predicted transfer-free energies of mammalian TM2 and TM6 suggest the modifications within these regions could potentially compromise the efficiency of the cotranslational folding of mammalian GnRHRs.

### Impact of polar substitutions on the cotranslational folding of TMs 2 and 6

**Figure 5.**
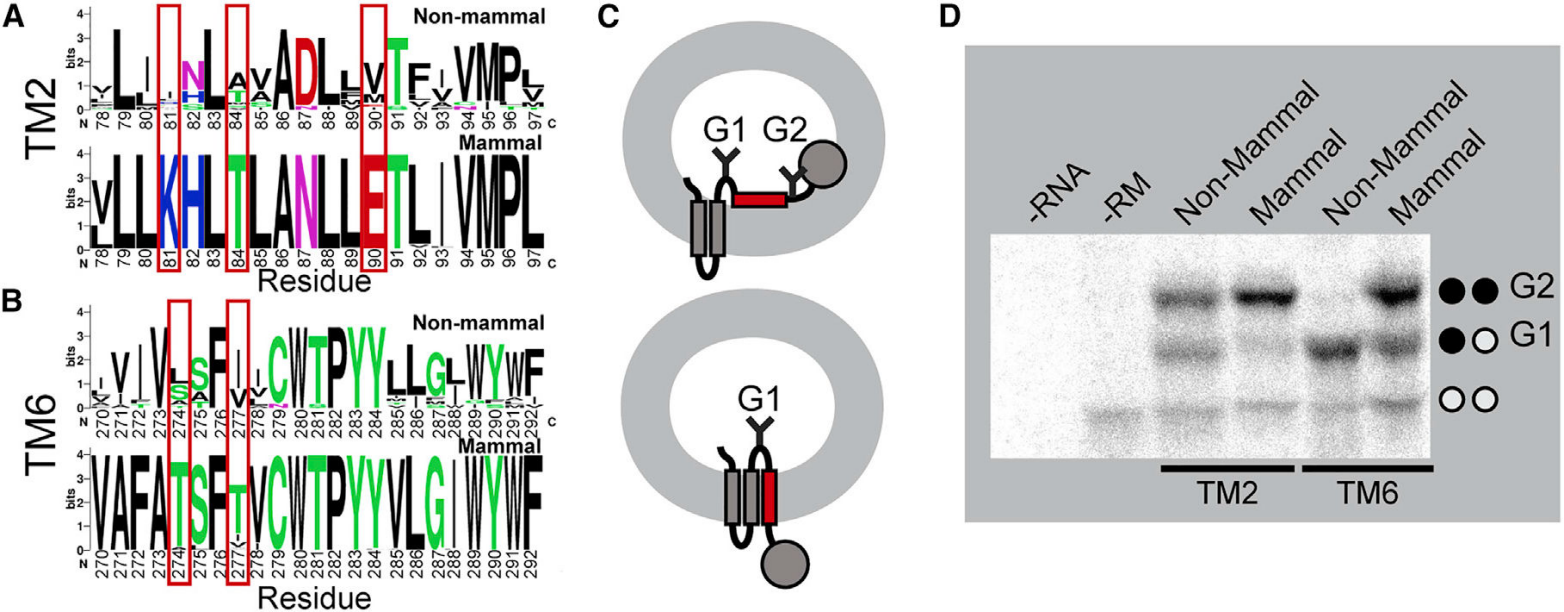
Translocon-mediated membrane integration of TMs 2 and 6 Differences in the sequences of TM2 and TM6 are analyzed in relation to differences in their efficiency of translocon-mediated membrane integration. (A and B) Logo plots depict the most common amino acid at each position within the non-mammalian (top) and mammalian (bottom) forms of (A) TM2 and (B) TM6. The positions of polar substitutions are indicated with a red box. Residue numbers are indexed to the human receptor. (C) A cartoon depicts the manner in which the translocon-mediated membrane integration of the guested TM domain within chimeric Lep proteins impacts their glycosylation. A failure of the guest TM domain to undergo translocon-mediated membrane integration results in the glycosylation of two residues (top), whereas the membrane integration of the guest domain results in a single glycosylation (bottom). (D) A representative SDS-PAGE image shows chimeric Lep proteins containing the mammalian or non-mammalian consensus sequences for TM2 and TM6, translated in canine rough microsomes. Negative control reactions lacking RNA (first lane) or containing RNA but lacking microsomes (second lane) are shown for reference. The positions of the untargeted (no glycans), singly glycosylated (G1), and doubly glycosylated (G2) forms of the protein are indicated for reference. See also Table S2.

To further explore these TM domains, we constructed logo plots that depict the most common amino acids found at each position within TM2 and TM6 of mammalian and non-mammalian GnRHRs. The sequences of the non-mammalian TM domains are more diverse than those of the mammalian receptor (Figures 5A and 5B), which reflects the increased evolutionary distance between the non-mammalian sequences (Figure 3). Nevertheless, a comparison of the most common amino acids at each position reveals the heightened transfer-free energies of mammalian GnRHRs primarily arise from three polar substitutions in TM2 and two polar substitutions in TM6 (Figures 5A and 5B). To assess the impact of these substitutions on the fidelity of stage I folding, we compared the translocon-mediated membrane integration of the consensus versions of the mammalian and non-mammalian TM domains. Briefly, a series of chimeric leader peptidase (Lep) proteins containing each TM domain of interest was produced by *in vitro* translation in canine rough microsomes. The membrane integration efficiency of each TM domain can then be inferred from the glycosylation state of Lep; membrane integration of the TM domain results in a single glycosylation whereas passage into the lumen generates two glycosyl modifications (Figure 5C). The Lep protein containing the non-mammalian TM2 is produced as a mix of both glycoforms (Figure 5D). In contrast, the doubly glycosylated form predominates for the Lep protein containing the mammalian TM2 (Figure 5D), which demonstrates that the increased polarity of the mammalian TM2 compromises its recognition by the translocon. Similarly, the membrane integration of the non-mammalian form of TM6 appears to be significantly more efficient than that of the corresponding mammalian form (Figure 5D). Although the apparent transfer-free energies associated with the translocon-mediated membrane integration of these helices slightly deviate from the predicted values (Table S2), these estimates correctly predict the manner in which these mutations should impact the efficiency of membrane integration. It should be noted that the orientation of these helices is inverted in the context of the Lep protein, which could also lead to deviations in membrane integration efficiency in the context of the full-length receptor. Nevertheless, both computational predictions (Figure 4B) and biochemical experiments (Figure 5D) suggest the increase in the polarity of TM2 and TM6 of mammalian GnRHRs decreases the efficiency of translocon-mediated membrane integration.

### Structural context of polar residues and their impacts on PME

**Figure 6.**
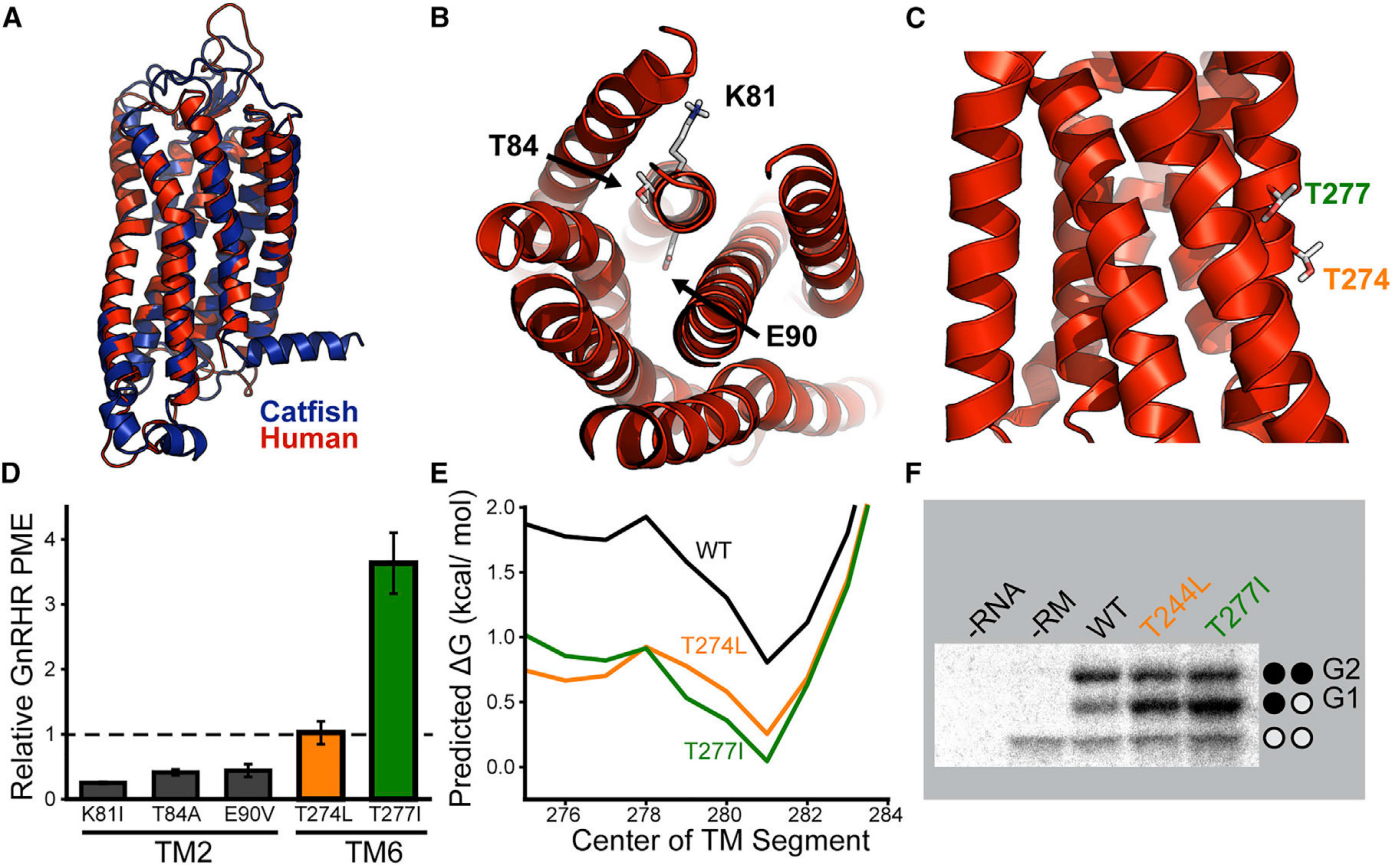
Structural context and proteostatic impacts polar residues within TMs 2 and 6 (A) Structural homology models of human (red) and catfish (blue) GnRHRs are overlaid for reference. (B) A cutaway of the human GnRHR homology model shows that the polar residues of interest within TM2 (K81, T84, and E90) are buried within the core of the hGnRHR protein. (C) A side view shows that the polar residues of interest within TM6 of human GnRHR appear to be projected into the membrane core. (D) Polar side chains within TMs 2 and 6 were replaced with the most common hydrophobic residues found within non-mammalian GnRHRs, and the effects of these substitutions one the plasma membrane expression (PME) of human GnRHR was measured in HEK293T cells by flow cytometry. A bar graph depicts the mean fluorescence intensity associated with the surface immunostaining of each variant normalized relative to that of WT human GnRHR. Values reflect the average of three biological replicates, and error bars reflect the standard deviation. (E) The TM6 region of WT (black), T244L (orange), and T277I (green) hGnRHR was scanned with 23-residue windows using the ΔG predictor, and the predicted free energy difference associated with the translocon-mediated membrane integration of the nascent chain is plotted as a function of the central position of each segment. (F) A representative SDS-PAGE image shows chimeric Lep proteins containing variants of hGnRHR TM6, translated in canine rough microsomes. Negative control reactions lacking RNA (first lane) or containing RNA but lacking microsomes (second lane) are shown for reference. The positions of the untargeted (no glycans), singly glycosylated (G1), and doubly glycosylated (G2) forms of the protein are indicated for reference.

Logo plots show that several polar residues were introduced within TM2 and TM6 during the evolutionary adaptation of mammalian GnRHRs (Figures 5A and 5B). Although these mutations disrupt cotranslational folding (Figure 5D), it is possible that they also help to stabilize the structure of the folded receptor and/or enhance its function. To gain insights into the structural context of these residues, we constructed comparative models of both the cGnRHR and hGnRHR receptors. With the exception of the C-terminal tail of cGnRHR, both forms of the receptor have a similar architecture (Figure 6A, Cα root-mean-square deviation [RMSD], 2.95 Å). The model of hGnRHR reveals that each of the three polar substitutions within TM2 occurs at positions that are buried within the protein core (Figure 6B). Thus, these side chains form tertiary contacts that may support the folding and/or function of mammalian GnRHR. Indeed, E90 is believed to form a key salt bridge that stabilizes the native conformation (Houck et al., 2014; Jardón-Valadez et al., 2008). In contrast, the two polar substitutions within TM6 appear to have occurred at residues that are exposed to the hydrophobic core of the lipid bilayer (Figure 6C). Although a complex role of these side chains in the native conformational dynamics cannot be ruled out, their solvent exposure potentially suggests these substitutions may tune the efficiency of cotranslational folding without perturbing function.

If adaptive mutations primarily compromised the membrane integration of TMs 2 and 6, then substitutions that restore the hydrophobicity of these domains may restore receptor PME. We therefore characterized the effects of ancestral hydrophobic substitutions in TM2 and TM6. Our structural model of hGnRHR suggests the hydrophobicity of TM2 cannot be restored without generating tertiary packing defects in the native structure (Figure 6B). It is therefore unsurprising that these mutations reduce PME of hGnRHR (Figure 6D); any changes in membrane integration may be offset by a loss of stability. In contrast, hydrophobic substitutions at surface-exposed residues in TM6 appear to be well-tolerated (Figure 6D). Although T274L has no impact on PME, T277I enhances the PME of GnRHR 3.6- ± 0.5-fold (Figure 6D). Moreover, the distinct proteostatic effects of these mutations track with their impacts on the efficiency of membrane integration. T227 is buried deeper within the membrane core, and the T277I mutation is therefore predicted to enhance membrane integration more than T274L (Figure 6E). Consistent with these predictions, we find that, in the context of chimeric Lep proteins, the membrane integration of the T277I variant of TM6 is slightly more efficient (ΔG_app_ = −0.29 ± 0.01 kcal/mol) than that of the T274L variant (ΔG_app_ = −0.22 ± 0.04 kcal/mol) (Figure 6F). Thus, our results suggest that a subtle increase in the hydrophobicity of TM6 is sufficient to partially restore the PME of hGnRHR. It is unclear why these variants exhibit such striking differences in proteostasis given that the underlying mutations have similar effects on membrane integration. Nevertheless, such differences could potentially arise from perturbations of the interhelical interactions that mediate topogenesis in the context of the full-length receptor (Öjemalm et al., 2012). In conjunction with *in vitro* translation measurements (Figure 5D), this observation implies that the enhanced cotranslational misfolding of mammalian TM6 contributes to the attenuated PME of the mammalian receptors.

### Functional impact of the T277I substitution

**Figure 7.**
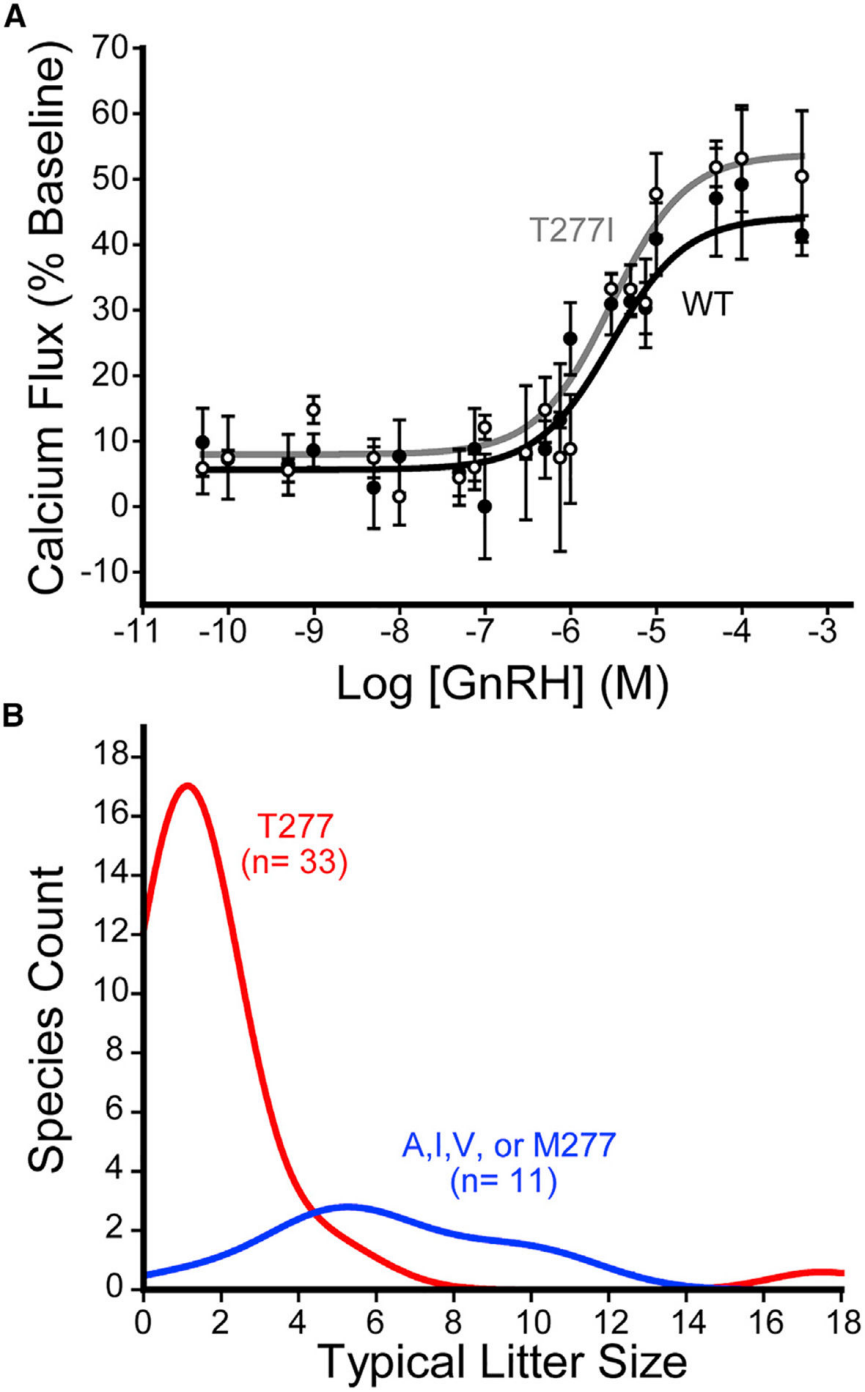
Hydrophobicity of residue 277 in relation to GnRHR function and mammalian litter size (A) The activation of WT and T277I hGnRHR was measured in response to varying doses of human gonadotropin releasing hormone (GnRH) in HEK293T cells. Cells transiently expressing each receptor were stimulated with GnRH, and signaling was measured by the change in the fluorescence intensity of a cytosolic calcium reporter. The average fluorescence intensities of cells expressing WT (●) or T277I (○) from three technical replicates are normalized relative to baseline and plotted against the corresponding concentration of hormone. Error bars reflect the SD from three technical replicates. Curves reflect the fit of the WT (black) and T277I (gray) data to a single-site binding model. (B) A histogram depicts the distribution of litter sizes for mammals bearing a polar (red) or hydrophobic (blue) side chain at residue 277. See also Table S3.

Our results collectively reveal that the enhanced polarity of TM6 compromises the cotranslational folding and expression of mammalian GnRHRs, and the reversion of a single surface residue to its ancestral hydrophobic side chain (T277I) is sufficient to partially recover PME. Nevertheless, it is possible that this side chain was introduced to support GnRHR function. To determine whether this residue is important for GnRHR signaling, we compared the activity of wild-type (WT) and T277I hGnRHRs. Briefly, cells transiently expressing WT or T277I GnRHR were titrated with gonadotropin-releasing hormone (GnRH), and the receptor activation was indirectly measured by the magnitude of the resulting cytosolic calcium flux. Cells expressing these receptors exhibit robust response to GnRH, which demonstrates both forms of the receptor are active (Figure 7A). Although T277I GnRHR is much more abundant at the plasma membrane (Figure 6D), it generates a calcium flux that is only slightly greater than those generated by WT (Figure 7A). This observation potentially suggests the activation of either of these overexpressed receptors is adequate to maximize the magnitude of the downstream signaling response in the context of HEK293T cells overexpressing each receptor. Nevertheless, the fitted EC_50_ values for WT (0.61 ± 0.38 μM) and T277I (0.23 ± 0.18 μM) were found to be statistically indistinguishable (Figure 7A). Therefore, these findings demonstrate that T277 is not essential for hGnRHR function. Given that this non-essential polar side chain negatively impacts cotranslational folding and PME, our collective observations suggest that the evolved polarity of this segment serves to tune the PME of mammalian GnRHRs.

### Sequence variations in relation to reproductive outcomes

Evolutionary variations in PME of GnRHR should have a direct influence on GnRH signaling, which may alter reproductive outcomes. Our results suggest that the hydrophobicity of residue 277 modulates the PME of GnRHR. To determine whether natural variation at this position coincides with differences in reproductive outcomes, we compared the hydrophobicity of the side chain at this position to litter size among 44 mammalian species. Species with a hydrophobic residue at this position have significantly larger litters than those with a hydrophilic residue (Figure 7B, Mann-Whitney p = 1.7 × 10^−5^), suggesting that the polarity of TM6 is associated with reproductive traits at the organismal level. Moreover, of the polar residues within TM2 and TM6, only residue 277 exhibits appreciable variation in hydrophobicity among the mammalian forms of the receptor (Figures 5A and 5B). These observations potentially suggest that modifications at T277, and their effects on the PME of GnRHR, may have played a direct role in the optimization of reproductive fitness.

## DISCUSSION

Previous investigations of the evolution of mammalian GnRHRs have suggested that their activity has been downregulated through a series of mutations that enhance their propensity to misfold (Janovick et al., 2003; Lin et al., 1998; Leaños-Miranda et al., 2003). In this work, we followed up on these investigations in order to assess the molecular basis for this evolved instability. We first quantitatively confirm previous findings (Janovick et al., 2006; Conn et al., 2006b) suggesting the expression of catfish GnRHR is robust relative to mammalian GnRHRs (Figure 1). To identify mutations that contributed to changes in GnRHR proteostasis, we then measured the impact of various sequence modifications on the PME of cGnRHR. Our results provide additional evidence that the truncation of the C-terminal tail results in a striking reduction in PME (Figure 2A) (Lin et al., 1998). Consistent with recent observations in other helical membrane proteins (Sun and Mariappan, 2020), we show that this truncation compromises the translocon-mediated membrane integration of TM7 (Figure 2C). Additionally, we find that the TM domains of mammalian GnRHRs are more polar than their non-mammalian counterparts (Figures 3 and 4), and this compromises the translocon-mediated membrane integration of two other TM domains (Figure 5). In most cases, the net contributions of individual side chains to receptor fitness are likely to be complex and to include impacts on both expression and function. Nevertheless, we identified two polar side chains within TM6 that lack tertiary interactions (Figure 6C) and show that restoring the hydrophobicity to one of these residues enhances the membrane integration in a manner that coincides with a 3.6-fold increase in PME with no impact on activation (Figures 6D, 6F, and 7A). This modification is likely relevant to the evolutionary trajectory of GnRHR considering the mouse receptor has a hydrophobic residue at this position (V276) and exhibits an enhanced PME relative to the human receptor (Figure 1). Indeed, the hydrophobicity of this residue is associated with striking variations in mammalian litter sizes (Figure 7B; Table S3). We note that, although the C-terminal truncation should uniformly decrease the fidelity of topogenesis of all mammalian receptors, the polarity of each TM domain varies widely among the mammalian receptors (Figure 4B). Thus, modifications to the efficiency of translocon-mediated cotranslational folding may have directly facilitated the adaptive differentiation of mammalian receptors.

Our findings provide additional evidence to suggest the activity of mammalian GnRHRs has been tuned through modulation of GnRHR folding rather than through transcriptional modifications. This is perhaps surprising given the metabolic cost of protein synthesis. Nevertheless, we believe this outcome is reasonable in light of certain evolutionary considerations. It should first be noted that the length of the open reading frame of GnRHR is roughly six times that of its promoter (Schang et al., 2012). Considering most coding variants are destabilizing, there are likely to be far more coding variants that decrease the PME of the receptor relative to the number that would simply decrease its transcription. If selection pressures simply favored attenuated GnRHR signaling, then it is perhaps most probable this would arise from mutations that destabilize the native GnRHR structure. Consistent with the observed proteostatic patterns (Figure 1), such mutations would result in both a decreased PME and an increased accumulation of the receptor within the secretory pathway. Nevertheless, the observed changes in net proteostasis may also include mutagenic effects on various cellular processes beyond those that are typically associated with protein quality control. Although the synthesis of hormone receptors that are destined to remain within the secretory pathway is energetically wasteful, it is unclear whether the biosynthesis of misfolded GnRHRs necessarily imposes a significant fitness burden in this case, as this receptor is only expressed at moderate levels within the pituitary gland according to the human protein atlas (humanproteinatlas: GNRHR). Thus, it seems plausible that various destabilizing mutations that fixed in mammals provided the gain in reproductive fitness that out-weighs the metabolic costs associated with the synthesis and degradation of misfolded receptors.

### Limitations of study

There are several caveats to these investigations. First, it should be noted that epistatic interactions between some of these mutations may alter their effects on PME in the context of mammalian receptors. Furthermore, we cannot measure the PME of these receptors in the context of their native environment within the pituitary gland of each animal. It is likely that the magnitude of these proteostatic effects is distinct in the context of the native proteostasis networks that typically support GnRHR biogenesis. Nevertheless, to our knowledge, mammalian GnRHRs are the only class A GPCRs that completely lack helix 8 and/or a C-terminal tail (Millar, 2005). Given that that the mechanism of the translocon is highly conserved, we suspect this truncation and the increased polarity of TM2 and TM6 are likely to compromise the efficiency of cotranslational GnRHR folding in any cellular context (Denks et al., 2014). Indeed, the hydrophobicity of TM domains is also known to be a critical factor that governs the expression of membrane proteins in *E. coli* (Niesen et al., 2017; Marshall et al.; 2016). Based on these considerations, it seems likely that both the C-terminal truncation and the enhanced the polarity of the TM domains of GnRHR are likely to have contributed to evolutionary modifications to the PME and net activity of mammalian GnRHRs. GnRHR represents a unique evolutionary model because the nature of the selection pressure that shaped its evolution is somewhat understood. Absent similar information, it is unclear how much these proteostatic factors may have shaped the evolution of other membrane proteins. Nevertheless, it stands to reason that changes in the fidelity of translocon-mediated membrane integration must factor into the fitness effects of mutations in membrane proteins.

### Conclusions

Marginal conformational stability is an emergent property of naturally evolved proteins (Tokuriki and Tawfik, 2009; Bloom et al., 2006; Taverna and Goldstein, 2002). This instability has been previously attributed to the net-destabilizing effects of random mutations in conjunction with a general lack of selection pressure for hyper-stable proteins (Tokuriki and Tawfik, 2009). Our recent findings in the context of rhodopsin have shown how mutations in marginally hydrophobic TM domains can tune expression (Roushar et al., 2019; Penn et al., 2020). The natural exploitation of the thin energetic margins involved in cotranslational MP folding perhaps also explains why the hydrophobicity of rhodopsin’s TM domains have not been optimized to promote efficient biosynthesis (Roushar et al., 2019). However, the apparent malleability of cotranslational MP folding energetics does not come without costs, as TM domains are generally less tolerant of genetic variation and mutations within TM domains give rise to numerous genetic diseases (Marinko et al., 2019; Schlebach and Sanders, 2015; Telenti et al., 2016).

Although it is likely that the instability of GnRHR emerged as a result of genetic drift, the net variation in PME resulting from these mutations was likely constrained by adaptive changes in reproductive fitness. Mammals, which have fewer offspring at higher metabolic cost, may require less GnRHR activity than non-mammals to maintain reproduction (Janovick et al., 2006). The loss of the C-terminal tail and the increased polarity of the TM domains, although deleterious to folding and PME, may have been tolerated due to an attenuated reliance on GnRH signaling. Nevertheless, it is certainly possible that the variation in GnRHR PME arising from the truncation of the C terminus and/or the incorporation of polar residues into TM2 and TM6 played an active role in the optimization of mammalian reproductive fitness. Together, these observations provide insights into the molecular mechanisms of membrane protein evolution.

## STAR★METHODS

### RESOURCE AVAILABILITY

#### Lead contact

Further information and requests for resources and reagents should be directed to and will be fulfilled by the lead contact, Jonathan Schlebach (jschleba@indiana.edu).

#### Materials availability

Plasmids generated for this paper are available from the lead contact upon request.

#### Data and code availability

- Flow cytometry, functional, phylogenetic, and *in vitro* translation data and structural models are available in the Dryad Digital Repository and are publicly available as of the date of publication. The DOI is listed in the Key Resources Table.
- This paper does not report original code.
- Any additional information required to reanalyze the data reported in this paper is available from the lead contact upon request.

### EXPERIMENTAL MODEL AND SUBJECT DETAILS

HEK293T cells were obtained from ATCC. Cells were grown in Dulbecco’s Modified Eagle’s medium (DMEM, GIBCO, Grand Island, NY) supplemented with 10% fetal bovine serum (FBS, GIBCO, Grand Island, NY), 0.5% penicillin (GIBCO, Grand Island, NY), and 0.5% streptomycin (GIBCO, Grand Island, NY) at 37°C and 5.0% CO_2_.

### METHOD DETAILS

#### Plasmid Preparation and Mutagenesis

A series of pcDNA5 FRT expression vector containing various GnRHR cDNAs containing an N-terminal influenza hemaglutinin (HA) epitope were used for the transient expression of GnRHR variants. GnRHR cDNAs in this vector are followed by an internal ribosome entry site (IRES) and eGFP sequence, which generates bicistronic GFP expression in positively transfected cells. Vectors containing GnRHRs from various species were generated using In-Fusion HD Cloning (Takara Bio, Shiga, Japan). Mutations were introduced by site-directed mutagenesis with PrimeSTAR HS DNA Polymerase (Takara Bio, Shiga, Japan), and truncations were generated by In-Fusion HD cloning. To adapt these constructs for functional experiments, the HA epitope was deleted to minimize interferences with ligand binding, and the IRES eGFP sequence was deleted to prevent interference of eGFP with fluorescence measurements.

A previously described pGEM expression vector containing either modified leader peptidase (Lep) or modified cGnRHR cDNA was used for *in vitro* translation (Hessa et al., 2007). For chimeric Lep proteins, TM domains of interest were cloned into the H-segment position within the Lep gene using directional cloning at the *Spe*I and KpnI restriction sites. cGnRHR cDNA was inserted into the pGEM vector using the NEBuilder HiFi DNA Assembly Kit (New England Biolabs, Ipswich, MA). cGnRHR was modified for *in vitro* translation by removing a native glycosylation sequence in the C-terminal tail (N346Q) and inserting a glycosylation sequence at the C terminus. Mutations were introduced by site-directed mutagenesis with PrimeSTAR HS DNA Polymerase. All plasmids were prepared with the Endotoxin-Free Zymopure Midiprep or Miniprep Kit (Zymo Research, Irvine, CA).

#### *In vitro* Translation of cGnRHR and Chimeric Lep Proteins

Messenger RNA (mRNA) was generated using the RiboMAX SP6 kit (Promega, Madison, WI) and purified either by TRIzol extraction (Ambion, Waltham, MA) or with an RNA Clean & Concentrator-5 Kit (Zymo Research, Irvine, CA). mRNA samples were then translated using rabbit reticulocyte lysate (Promega, Madison, WI) supplemented with canine rough microsomes (tRNA probes, College Station, TX), and EasyTag ^35^S-labeled methionine (PerkinElmer, Waltham, MA). Translation was carried out at 30°C for 60 minutes. Reactions were diluted 1:4 in 1X SDS-PAGE loading buffer and separated on a 12% SDS-PAGE gel. Gels were then dried, exposed overnight on a phosphor imaging plate (GE Healthcare, New York, NY), and imaged on a Typhoon Imager (GE Healthcare, New York, NY). For chimeric Lep proteins, the ratio of singly (*G1*) to doubly (*G2*) glycosylated Lep protein was quantified by densitometry using ImageJ software. The *G1:G2* ratio represents an apparent equilibrium constant (*K_app_*) for the transfer of the H-segment from the translocon to the membrane, as previously described. Apparent transfer free energy values for the H-segments were calculated using the following equation:

$$\Delta G_{app}=-RT\;\ln(K_{app})=-RT\;\ln\left(\frac{G1}{G2}\right)$$

where Δ*G_app_* represents the apparent free energy for the transfer of the H-segment into the membrane, *R* represents the universal gas constant, *T* represents the temperature, *K_app_* represents the apparent equilibrium constant for the transfer of the H-segment from the translocon into the membrane, *G1* represents the intensity of the singly glycosylated band and *G2* represents the intensity of the doubly glycosylated band, as was previously described (Hessa et al., 2005). Reported transfer free energy values represent the average values from three experimental replicates.

#### Cellular GnRHR Expression Measurements

To quantitatively measure the cellular trafficking of GnRHR variants, plasma membrane and intracellular GnRHRs were differentially immunostained and analyzed by flow cytometry, as described previously (Schlebach et al., 2015). GnRHR variants were transiently expressed in HEK293T cells using Lipofectamine 3000 (Invitrogen, Carlsbad, CA). Two days after transfection, the cells were washed with 1X phosphate-buffered saline (GIBCO, Grand Island, NY) and harvested with TrypLE Express protease (GIBCO, Grand Island, NY). Plasma membrane GnRHRs were then immunostained for 30 minutes in the dark with a DyLight 550-conjugated anti-HA antibody (Invitrogen, Carlsbad, CA). Cells were fixed and permeabilized using a Fix and Perm kit (Invitrogen, Carlsbad, CA), and washed twice with 2% fetal bovine serum in phosphate-buffered saline (wash buffer). Intracellular GnRHRs were then immunostained for 30 minutes in the dark using an Alexa Fluor 647-conjugated anti-HA antibody (Invitrogen, Carlsbad, CA). Cells were washed twice in order to remove excess antibody prior to analysis of cellular fluorescence profiles. Fluorescence profiles were analyzed on a BD LSRII flow cytometer (BD Biosciences, Franklin Lakes, NJ). Forward and side scatter measurements were used to set a gate for intact single cells. eGFP intensities (488 nm laser, 530/30 nm emission filter) were then used to set a gate for positively-transfected cells. DyLight 550 (561 nm laser, 582/15 nm emission filter) and Alexa Fluor 647 (640 nm laser, 670/30 nm emission filter) intensities were then calculated for several thousand positively-transfected single cells within each biological replicate. Data were analyzed using FlowJo software (Treestar, Ashland, OR). Characterizations of GnRHR variant expression levels were carried out with at least three biological replicates each.

#### Functional Measurements of GnRHRs

GnRHR activity was measured in HEK293T cells by monitoring cytosolic calcium fluxes that occurred in response to gonadotropin-releasing hormone (GnRH, Sigma Aldrich, St. Louis, MO). GnRHR variants were transiently expressed in HEK293T cells using Lipofectamine 3000 (Invitrogen, Carlsbad, CA). Two days after transfection, the cells were washed with 1X phosphate-buffered saline (GIBCO, Grand Island, NY) and harvested with TrypLE Express (GIBCO, Grand Island, NY), then re-plated in 96-well plates (Corning, Big Flats, NY) coated with poly-D-lysine (GIBCO, Grand Island, NY) at a density of 60,000 cells per well. Cells were then dosed the following day and assayed using the FLIPR Calcium 6-QF Assay Kit (Molecular Devices, San Jose, CA) according to the manufacturer’s protocol. The fluorescence intensities of cells incubated in the calcium-sensitive FLIPR dye was measured for thirty seconds prior to dosing with GnRH using a Synergy Neo2 microplate reader (BioTek, Winooski, VT) using an excitation wavelength of 485/20 nm and an emission filter at 525/10 nm. Directly after dosing the cells, the change in fluorescence was measured for six minutes. The percent calcium flux under each condition was calculated for each well using the following equation:

$$\text{Percent Calcium Flux}=\frac{M-B}{B}\times 100$$

where *M* is the maximum fluorescence value for the calcium flux and *B* is the baseline signal as was determined from by averaging the fluorescence intensity before ligand addition. EC_50_ values were determined by fitting titrations to the following function:

$$Y=A+\frac{B-A}{1+m\cdot 10^{C-x}}$$

where *Y* is the percent calcium flux, *A* is the minimal curve asymptote, *B* is the maximal curve asymptote, *m* is the slope of the transition region, *C* is the logarithm of the EC_50_, and *x* is the logarithm of the GnRH concentration (Motulsky and Christopoulos, 2003). Reported EC_50_ values represent the average from three biological replicates.

#### Selection and Analysis of GnRHR Sequences

59 GnRHR sequences from different species were collected from the NCBI (https://www.ncbi.nlm.nih.gov) and Uniprot (https://www.uniprot.org) databases. Humans have only one type of GnRHR (GnRHR-I), while other species may have multiple types (Pawson et al., 2003; Tello et al., 2008). Sequences selected for phylogenetic analysis were therefore limited to those annotated as GnRHR-I in order to analyze trends across species.

A phylogenetic tree was generated from these sequences using MEGA7 software (megasoftware.net). A sequence alignment was generated using the MUSCLE alignment tool with default settings. This alignment was then used to construct a Maximum Likelihood tree (Kumar et al., 2016). The positions of nascent TM domains within each sequence were then identified from energetic minima generated with a window scan function within the ΔG predictor, which sums depth-dependent free energies associated with the transfer of amino acids from the translocon to the ER membrane (http://dgpred.cbr.su.se/) (Hessa et al., 2005). The ΔG predictor was then used to calculate the free energy difference associated with the translocon-mediated membrane integration of each putative TM domain (Hessa et al., 2007). The phylogenetic tree and ΔG prediction data were then uploaded to the Interactive Tree of Life (https://itol.embl.de), where the ΔG predictions were displayed as color gradients on the phylogenetic tree (Letunic and Bork, 2016).

To generate logo plots, the GnRHR-I sequences were first aligned in ClustalOmega (Sievers et al., 2011). The positions of the TM domains within the hGnRHR sequence were determined by the ΔG predictor, and the transmembrane domains in other species were then identified by the corresponding positions in the alignment. Sequence logo plots were then generated for each transmembrane domain in the mammalian and non-mammalian sequences using the WebLogo application (https://weblogo.berkeley.edu/logo.cgi) (Crooks et al., 2004).

Litter size data was collected for 44 species corresponding to the mammalian GnRHR-I sequences. The average litter size or the middle of a range was used as the typical litter size. For species where twins or multiples are rare, the typical litter size was set to one. The residue equivalent to T277 in hGnRHR was determined for each mammalian GnRHR-I sequence by an alignment in ClustalOmega (Sievers et al., 2011).

#### Structural Modeling

Comparative models of the human and catfish forms GnRHR were generated using multi-template comparative modeling in Rosetta. The GnRHR sequence was first aligned with sequences for 34 GPCR crystal structures obtained from GPCRdb (http://www.gpcrdb.org) (Isberg et al., 2016). Manual adjustments were then made to account for well-known conserved residues in loop regions and TM domains (Bender et al., 2019). OCTOPUS was used to define the TM domains, and the two disulfide bonds were defined manually (Millar et al., 2004; Viklund and Elofsson, 2008). To generate a model of GnRHR in the inactive state, the sequences were threaded onto the antagonist-bound structures of several other Class A Group β GPCRs including the human OX2 orexin receptor (HCRTR2, PDB 4S0V, 2.5 Å), human OX1 orexin receptor (HCRTR1, PDB 4ZJC, 2.8 Å), human endothelin receptor type B (EDNRB, PDB 5X93, 2.2 Å), and human neuropeptide Y receptor Y1 (NPY1R, PDB 5ZBQ, 2.7 Å). Threading was completed using the *partial thread* application in RosettaCM (Song et al., 2013). 1,000 models were then generated using the *hybridize* application in RosettaCM and the TM domains were relaxed using a set of optimized RosettaMembrane weights that were modified from the Talaris scoring function (Duran and Meiler, 2018). The models with the lowest Rosetta energy score were used for structural analysis.

### QUANTIFICATION AND STATISTICAL ANALYSIS

Statistical information can be found in figure legends and Method Details. Mann-Whitney tests were used to calculate p values, and p < 0.05 were considered statistically significant. Flow cytometry data were analyzed in FlowJo software (Treestar, Ashland, OR), and *in vitro* translation data were analyzed in ImageJ software. Functional data, litter size data, and ΔG predictions were analyzed in Origin software (OriginLab, Northampton, MA).

## Supplementary Material

1

## Figures and Tables

**Table:** KEY RESOURCES TABLE

REAGENT or RESOURCE	SOURCE	IDENTIFIER
Antibodies		
DyLight 550-conjugated anti-HA antibody	Invitrogen	Cat# 26183-D550, RRID AB_2533052
Alexa Fluor 647-conjugated anti-HA antibody	Invitrogen	Cat# 26183-A647, RRID AB_2610626
Chemicals, peptides, and recombinant proteins		
In-Fusion HD	Takara Bio	Cat# 638918
SpeI	New England Biolabs	Cat# R3133S
KpnI	New England Biolabs	Cat# R0142S
EasyTag 35S-labeled methionine	PerkinElmer	Cat# NEG709A500UC
Lipofectamine 3000	Invitrogen	Cat# L3000008
TrypLE Express protease	GIBCO	Cat# 12605010
FIX & PERM Cell Permeabilization Kit	Invitrogen	Cat# GAS003
Gonadotropin-releasing hormone (GnRH)	Sigma-Aldrich	Cat# L8008
Poly-D-lysine	GIBCO	Cat# A3890401
10X PBS	GIBCO	Cat# 70011-044
DMEM	GIBCO	Cat# 11965-092
Opti-MEM	GIBCO	Cat# 31985-062
1X PBS	GIBCO	Cat# 20012-050
Penicillin/streptomycin	GIBCO	Cat# 15140-122
FBS	Corning	Cat# 35-010-CV
TRIzol	Ambion	Cat# 15596018
PrimeSTAR HS DNA polymerase	Takara Bio	Cat# R010B
Canine rough microsomes	tRNA Probes	N/A
NEBuilder HiFi DNA Assembly Cloning Kit	New England Biolabs	E5520S
Critical commercial assays		
Zymopure Miniprep Kit	Zymo Research	Cat #D4208T
Zymopure Midiprep Kit	Zymo Research	Cat# D4200
RiboMAX Large Scale RNA Production System SP6	Promega	Cat# P1290
FLIPR Calcium 6-QF Assay Kit	Molecular Devices	Cat# R8192
RNA Clean & Concentrator-5	Zymo Research	Cat# R1016
Rabbit Reticulocyte Lysate, Nuclease-Treated	Promega	Cat# L4960
Deposited data		
Flow cytometry, functional, phylogenetic, and IVT/Lep data; structural models	This paper	https://doi.org/10.5061/dryad.dncjsxkvs
Experimental models: cell lines		
HEK293T	ATCC	Cat# CRL-11268
Recombinant DNA		
Plasmid: modified pcDNA5 with cGnRHR WT	This paper	N/A
Plasmid: modified pGEM Lep with GnRHR non-mammalian TM2 consensus sequence	This paper	N/A
Plasmid: modified pGEM Lep with GnRHR mammalian TM2 consensus sequence	This paper	N/A
Plasmid: modified pGEM Lep with GnRHR non-mammalian TM6 consensus sequence	This paper	N/A
Plasmid: modified pGEM Lep with GnRHR mammalian TM6 consensus sequence	This paper	N/A
Plasmid: modified pcDNA5 with hGnRHR K81I	This paper	N/A
Plasmid: modified pcDNA5 with hGnRHR T84A	This paper	N/A
Plasmid: modified pcDNA5 with hGnRHR E90V	This paper	N/A
Plasmid: modified pcDNA5 with hGnRHR T274L	This paper	N/A
Plasmid: modified pcDNA5 with hGnRHR T277I	This paper	N/A
Plasmid: modified pGEM Lep with GnRHR mammalian TM6 T274L	This paper	N/A
Plasmid: modified pGEM Lep with GnRHR mammalian TM6 T277I	This paper	N/A
Plasmid: modified pGEM with cGnRHR N346Q	This paper	N/A
Plasmid: modified pGEM with cGnRHR N346Q, deletion of the C-terminal tail	This paper	N/A
Plasmid: modified pcDNA5 with cGnRHR C339A/C341A	This paper	N/A
Plasmid: modified pcDNA5 with cGnRHR, deletion of residues 352-379	This paper	N/A
Plasmid: modified pcDNA5 with cGnRHR, deletion of residues 329-379 (C-terminal tail)	This paper	N/A
Plasmid: modified pcDNA5 with hGnRHR WT	This paper	N/A
Plasmid: modified pcDNA5 with mGnRHR WT	This paper	N/A
Software and algorithms		
FlowJo	TreeStar	https://www.flowjo.com/solutions/flowjo
NCBI	National Center for Biotechnology Information	https://www.ncbi.nlm.nih.gov/
Uniprot	The Uniprot Consortium	https://www.uniprot.org/
MEGA7	Kumar et al., 2016	https://megasoftware.net/
Prediction of ΔG for TM Helix Insertion	Hessa et al., 2007	https://dgpred.cbr.su.se/index.php?p=home
Interactive Tree of Life	Letunic and Bork, 2016	https://itol.embl.de
Clustal Omega	Sievers et al., 2011	https://www.ebi.ac.uk/Tools/msa/clustalo/
WebLogo	Crooks et al., 2004	https://weblogo.berkeley.edu/logo.cgi
GPCRdb	Isberg et al., 2016	http://www.gpcrdb.org
OCTOPUS	Viklund and Elofsson, 2008	https://octopus.cbr.su.se/; https://topcons.net/
RosettaCM	Song et al., 2013	N/A
RosettaMembrane	Duran and Meiler, 2018	N/A
ImageJ	NIH	https://imagej.nih.gov/ij/
Origin	OriginLab	https://www.originlab.com/
Other		
Synergy Neo2 Microplate Reader	BioTek	N/A
Typhoon Imager	GE Healthcare	N/A
BD LSRII flow cytometer	BD Biosciences	N/A

## Highlights

- Mammalian GnRHRs tend to misfold and fail to traffic to the plasma membrane
- A C-terminal truncation compromises the membrane integration of TM7
- Polar residues in TM2 and TM6 also jeopardize membrane integration and folding
- The polarity of TM6 tracks with differences in reproductive phenotypes
