# Supplementary material for: Molecular basis for the evolved instability of a human G-protein coupled receptor
Source: Cell Rep. Author manuscript; Available in PMC 2022 Feb 23. (PMC8865034; doi:10.1016/j.celrep.2021.110046)
Supplement: 1 [file NIHMS1759315-supplement-1.pdf]

Cell Reports, Volume *37*

Supplemental information

Molecular basis for the evolved instability of a human G-protein coupled receptor

Laura M. Chamness, Nathan B. Zelt, Haley R. Harrington, Charles P. Kuntz, Brian J. Bender, Wesley D. Penn, Joshua J. Ziarek, Jens Meiler, and Jonathan P. Schlebach

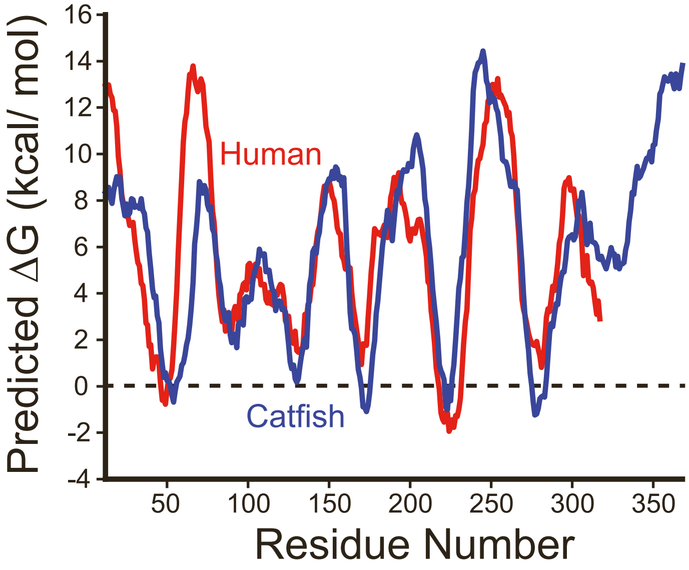

Figure S1. Topological energetics of human and catfish GnRHRs. Sequence-based predictions of the topological energetics of human GnRHR (red) are analyzed in relation to those of catfish GnRHR (blue). The predicted transfer free energy from the translocon to the ER membrane was predicted for each possible 23-residue segment within each protein were calculated using the ΔG predictor, and each predicted value was plotted as a function of the central residue of the segment. A ΔG value of 0 kcal/ mol, at which membrane integration is energetically neutral, is indicated with a dashed line. This figure pertains to the data shown in Figure 2.

Table S1. Predicted transfer free energies associated with the putative TM domains within GnRHRs.

		Predicted Transfer Free Energy (kcal/mol)							
**Species**	**Accession ID**	**TM1**	**TM2**	**TM3**	**TM4**	**TM5**	**TM6**	**TM7**	**7 TM Average**
Sebastes schlegelii	AFR51715.1	-0.291	0.825	0.906	1.105	-2.699	-0.554	0.876	0.024
Octopus vulgaris	Q2V2K5.1	-0.028	1.189	1.424	0.542	-2.913	-1.049	2.172	0.191
Xenopus tropicalis	NP_001107549.1	-0.637	-0.343	1.274	0.538	-3.978	-1.698	1.694	-0.450
Callorhinchus milii	NP_001279833.1	-1.615	2.202	1.382	-1.329	-2.228	-0.411	3.168	0.167
Dicentrarchus labrax	CAD11992.1	-0.816	0.825	0.906	0.663	-2.699	-0.323	1.166	-0.040
Danio rerio	XP_021323652.1	-0.621	1.556	-0.264	-0.995	-1.031	-1.108	4.667	0.315
Macrobrachium nipponense	AHB33640.1	0.957	0.78	2.604	-0.513	0.652	-2.433	-2.182	-0.019
Acanthopagrus schlegelii	AAV71128.1	-0.481	0.825	0.906	0.895	-3.319	-0.554	1.166	-0.080
Branchiostoma floridae	ACC68665.1	-0.883	0.546	-1.065	2.711	-3.374	-0.159	3.467	0.178
Odontesthes bonariensis	ABI75337.1	-1.482	1.45	-0.229	-1.171	-0.825	-1.1	4.821	0.209
Kryptolebias marmoratus	ABK88281.1	-0.311	0.826	-0.1	0.505	-2.422	-1.499	1.021	-0.283
Python bivittatus	XP_007437330.1	1.195	0.044	0.089	0.121	-3.593	-0.133	1.454	-0.118
Monopterus albus	ARS88253.1	-1.077	0.825	0.906	1.418	-2.699	-1.39	2.867	0.121
Cairina moschata	AGO01050.1	0.032	1.003	-0.425	-0.177	-3.323	-0.72	1.337	-0.325
Gallus gallus	AID62089.1	0.01	0.897	-0.425	0.082	-2.922	-0.634	1.785	-0.172
Ovis aries	NP_001009397.1	-0.854	2.352	1.334	0.677	-2.053	0.805	3.015	0.754
Bos Taurus	NP_803480.1	-0.854	2.165	1.334	0.677	-1.754	0.805	2.566	0.706
Canis lupus familiaris	NP_001003121.1	-1.053	2.352	1.334	0.305	-1.523	0.805	2.419	0.663
Bos mutus grunniens	Q19PY9.1	-0.854	2.165	1.334	0.677	-1.754	0.805	2.117	0.641
Sus scrofa	NP_999438.1	-0.646	2.352	1.334	0.341	-1.949	-0.748	2.566	0.464
Cavia porcellus	NP_001166428.1	-0.363	2.352	1.643	2.21	-2.785	-0.163	2.566	0.780
Trichosurus vulpecula	Q9TTI8.1	-0.267	2.456	0.655	-0.773	-1.129	-0.159	2.87	0.522
Delphinapterus leucas	XP_022417405.1	-0.38	2.352	1.179	0.306	-2.389	0.805	2.566	0.634
Ictidomys tridecemlineatus	XP_005331769.1	-0.188	2.352	1.334	0.942	-2.053	0.046	2.566	0.714
Neomonachus schauinslandi	XP_021558093.1	-0.38	2.497	1.334	0.305	-1.624	0.563	2.419	0.731
Phascolarctos cinereus	XP_020855496.1	-0.267	2.456	0.655	-0.134	-1.784	0.08	3.176	0.597
Odocoileus virginianus texanus	XP_020769734.1	-0.854	2.352	1.334	0.677	-1.754	0.805	2.566	0.732
Bubalus bubalis	ARJ58634.1	-0.854	2.165	1.334	0.677	-1.754	0.805	2.566	0.706
Capra hircus	NP_001272541.1	-0.854	2.352	1.334	0.677	-2.053	0.805	2.566	0.690
Equus caballus	NP_001075305.1	-0.38	2.352	1.334	0.535	-1.884	0.805	2.419	0.740
Felis catus	AFP97799.1	-0.38	2.316	1.334	0.389	-2.053	0.805	2.419	0.690
Sarcophilus harrisii	XP_003773267.1	-0.477	2.475	0.655	0.101	-1.947	0.08	2.87	0.537
Loxodonta Africana	XP_003415950.1	-0.767	2.165	1.832	0.764	-1.744	0.805	2.566	0.803
Dasypus novemcinctus	XP_004466930.1	-0.249	2.165	1.334	0.787	-1.901	0.303	2.566	0.715
Pteropus vampyrus	XP_011358956.1	-0.293	2.352	1.334	0.773	-1.481	0.805	2.566	0.865
Myotis lucifugus	XP_006095704.1	-0.636	2.352	1.334	0.639	-1.837	0.805	2.566	0.746
Physeter catodon	XP_007108070.1	-0.38	2.352	1.179	0.306	-2.504	0.805	2.566	0.618
Desmodus rotundus	XP_024433366.1	-0.72	2.352	1.643	0.251	-1.837	0.563	2.566	0.688
Neophocaena asiaeorientalis	XP_024603511.1	-0.38	2.352	1.179	0.306	-2.389	0.805	2.566	0.634
Pteropus alecto	XP_006916576.1	-0.38	2.352	1.334	0.773	-1.481	0.805	2.566	0.853
Canis lupus dingo	XP_025291626.1	-1.053	2.352	1.334	0.305	-1.523	0.805	2.419	0.663
Oryctolagus cuniculus	AAV48838.1	-0.091	2.352	1.334	0.431	-2.102	0.046	2.566	0.648
Rattus norvegicus	NP_112300.2	-0.361	2.352	1.461	-0.262	-2.053	0.037	2.566	0.534
Mus musculus	Q01776.1	-0.646	2.352	1.334	-0.262	-2.18	0.26	2.566	0.489
Heterocephalus glaber	XP_004849709.1	-0.401	2.352	1.03	1.501	-2.053	0.046	2.566	0.720
Mus caroli	XP_021019370.1	-0.646	2.352	1.334	-0.262	-2.18	0.26	2.566	0.489
Mus pahari	XP_021066838.1	-0.646	2.352	1.334	-0.29	-2.18	0.26	2.566	0.485
'Papio anubis	XP_003898885.1	-0.774	2.165	1.334	0.934	-2.053	0.805	2.566	0.711
Carlito syrichta	XP_008069339.1	-1.126	2.352	2.049	0.378	-2.053	0.805	2.419	0.689
Aotus nancymaae	XP_012328857.1	-0.38	2.165	0.655	0.934	-2.053	0.805	2.566	0.670
Microcebus murinus	XP_012616420.1	-0.453	2.352	1.334	0.764	-2.376	0.805	2.566	0.713
Theropithecus gelada	XP_025242022.1	-0.845	2.165	1.239	0.934	-2.053	0.805	2.566	0.687
Pan paniscus	XP_003815858.1	-0.774	2.165	1.334	0.934	-1.943	0.805	2.566	0.727
Macaca nemestrina	XP_011709114.1	-0.884	2.165	1.334	0.934	-2.053	0.805	2.566	0.695
Pan troglodytes	XP_526608.1	-0.774	2.165	1.334	0.934	-1.943	0.805	2.566	0.727
Pongo abelii	XP_024101999.1	-0.774	2.165	1.334	0.934	-2.053	0.805	2.566	0.711
Piliocolobus tephrosceles	XP_023087932.1	-0.774	2.165	1.334	0.934	-2.053	0.805	2.566	0.711
Otolemur garnettii	XP_003801017.1	-0.38	2.336	2.105	0.856	-2.247	0.673	2.566	0.844
Homo sapiens	AAB26287.1	-0.774	2.165	1.334	0.934	-1.943	0.805	2.566	0.727

**The data in this table corresponds to those shown in Figures 3 and 4.*

Table S2. Predicted versus measured apparent transfer free energies associated with TMs 2 & 6.

TM Segment	Predicted ΔG_{app} (kcal/ mol)†	Measured ΔG_{app} (kcal/ mol)‡
Non-mammalian TM2	0.73	0.15 ± 0.08
Mammalian TM2	2.42	0.85 ± 0.1
Non-mammalian TM6	-0.26	-1.38 ± 0.01
Mammalian TM6	0.81	0.23 ± 0.05

†Values were predicted from sequence using the ΔG Predictor.
‡Values reflect the average from three independent experimental replicates, and errors reflect the standard deviation.
**The data in this table pertain to Figure 5.*

Table S3. Litter size data associated with mammalian GnRHRs.

Species	Accession ID	Residue at Position 277	Typical Litter Size	Litter Size Reference
Ovis aries	NP_001009397.1	T	1.58	(Werner and Griebeler, 2011)
Bos Taurus	NP_803480.1	T	1	(Werner and Griebeler, 2011)
Canis lupus familiaris	NP_001003121.1	T	5.4	(Borge et al., 2011)
Bos mutus grunniens	Q19PY9.1	T	1	(Li et al., 2003; Werner and Griebeler, 2011)
Sus scrofa	NP_999438.1	I	5	(Wood and Barrett, 1979)
Cavia porcellus	NP_001166428.1	I	3.8	(Werner and Griebeler, 2011)
Trichosurus vulpecula	Q9TTI8.1	T	1	(Nowak, 2018)
Delphinapterus leucas	XP_022417405.1	T	1	(Tacutu et al., 2018; 'AnAge entry for Delphinapterus leucas')
Ictidomys tridecemlineatus	XP_005331769.1	I	10	(Cleary and Craven, 1994)
Neomonachus schauinslandi	XP_021558093.1	T	1	(Perrin et al., 2008)
Phascolarctos cinereus	XP_020855496.1	T	1	(Nowak, 2018)
Odocoileus virginianus texanus	XP_020769734.1	T	2	(Innes, 2013)
Bubalus bubalis	ARJ58634.1	T	1.38	(Werner and Griebeler, 2011)
Capra hircus	NP_001272541.1	T	1.5	(Werner and Griebeler, 2011)
Equus caballus	NP_001075305.1	T	1	(Werner and Griebeler, 2011)
Felis catus	AFP97799.1	T	4	(Root Kustritz, 2006)
Sarcophilus harrisii	XP_003773267.1	T	17.5	(Nowak, 2018)
Loxodonta Africana	XP_003415950.1	T	1	(Werner and Griebeler, 2011)
Dasypus novemcinctus	XP_004466930.1	M	4	(Hawthorne, 1994)
Pteropus vampyrus	XP_011358956.1	T	1	(Kunz et al., 2010)
Myotis lucifugus	XP_006095704.1	T	1	(Tacutu et al., 2018; 'AnAge entry for Myotis lucifugus')
Physeter catodon	XP_007108070.1	T	1	(Langer, 2008)
Desmodus rotundus	XP_024433366.1	T		(Tacutu et al., 2018; 'AnAge entry for Desmodus rotundus')
Neophocaena asiaeorientalis	XP_024603511.1	T	1	D. Wang. Personal communication. (April 15, 2020).
Pteropus alecto	XP_006916576.1	T	1.2	(Tacutu et al., 2018; 'AnAge entry for Pteropus alecto)
Canis lupus dingo	XP_025291626.1	T	4.5	(Hudson et al., 2016)
Oryctolagus cuniculus	AAV48838.1	I	5	(Werner and Griebeler, 2011)
Rattus norvegicus	NP_112300.2	V	9	(Timm, 1994)
Mus musculus	Q01776.1	V	7.4	(Gates, 1925)
Heterocephalus glaber	XP_004849709.1	I	11	(Yu et al., 2017)
Mus caroli	XP_021019370.1	V	6	(Zitnik et al., 1992)
Mus pahari	XP_021066838.1	V	6	(Agrawal, 2000)
Papio anubis	XP_003898885.1	T	1	(Harvey and Clutton-Brock, 1985)
Carlito syrichta	XP_008069339.1	T	1	(Tacutu et al., 2018; 'AnAge entry for Carlito syrichta')
Aotus nancymaae	XP_012328857.1	T	1	(Gozalo and Montoya, 1990)
Microcebus murinus	XP_012616420.1	T	2	(Tacutu et al., 2018; 'AnAge entry for Microcebus murinus')
Theropithecus gelada	XP_025242022.1	T	1	(Werner and Griebeler, 2011)

Pan paniscus	XP_003815858.1	T	1	(Werner and Griebeler, 2011)
Macaca nemestrina	XP_011709114.1	T	1	(Werner and Griebeler, 2011)
Pan troglodytes	XP_526608.1	T	1	(Werner and Griebeler, 2011)
Pongo abelii	XP_024101999.1	T	1	(Kelle et al., 2013)
Piliocolobus tephrosceles	XP_023087932.1	T	1	C. Chapman. Personal communication. (April 15, 2020).
Otolemur garnettii	XP_003801017.1	A	1	(Tacutu et al., 2018; 'AnAge entry for Otolemur garnettii')
Homo sapiens	AAB26287.1	T	1	(Harvey and Clutton-Brock, 1985)

*The data in this table pertain to Figure 7.

Supporting Citations from The Animal Ageing and Longevity Database. AnAge entries for *Carlito syrichta, Delphinapterus leucas, Desmodus rotundas, Microcebus murinus, Myotis lucifugus, Otolemur garnettii, and Pteropus alecto.*
